# How Do Emotions during Goal Pursuit in Weight Change over Time? Retrospective Computational Text Analysis of Goal Setting and Striving Conversations with a Coach during a Mobile Weight Loss Program

**DOI:** 10.3390/ijerph18126600

**Published:** 2021-06-19

**Authors:** Heather Behr, Annabell Suh Ho, Ellen Siobhan Mitchell, Qiuchen Yang, Laura DeLuca, Andreas Michealides

**Affiliations:** 1 Department of Integrative Health, Saybrook University, 55 W Eureka St, Pasadena, CA 91103, USA; hbehr@saybrook.edu; 2 Academic Research, Noom, 229 W 28th St., New York, NY 10461, USA; annabell@noom.com (A.S.H.); qiuchen@noom.com (Q.Y.); ldeluca@mail.yu.edu (L.D.); andreas@noom.com (A.M.); 3 Ferkauf Graduate School of Psychology, Yeshiva University, 1165 Morris Park Ave., Bronx, NY 10461, USA

**Keywords:** weight loss, goal pursuit, computational text analysis, mHealth, digital health, obesity, goal setting

## Abstract

During behavioral weight management, individuals reflect on their progress and barriers through goal pursuit (goal setting and goal striving). Emotions during goal pursuit are largely unknown, and previous investigations of emotions in weight management have primarily relied on self-report. In this retrospective study, we used a well-validated computational text analysis approach to explore how emotion words changed over time during goal setting and striving conversations with a coach in a mobile weight loss program. Linear mixed models examined changes in emotion words each month from baseline to program end and compared emotion words between individuals who set an overall concrete goal for the program (concrete goal setters) and those who set an overall abstract goal (abstract goal setters). Contrary to findings using self-report, positive emotion words were stable and negative emotion words significantly increased over time. There was a marginal trend towards greater negative emotion word use being associated with greater weight loss. Concrete goal setters used more positive words than abstract goal setters, with no differences in negative emotion words and weight loss. Implications include the possibility that individuals may need increasing support over time for negative emotions expressed during goal setting and striving, and concrete goals could boost positive emotion. Future research should investigate these possibilities.

## 1. Introduction

### 1.1. Emotions and Goal Pursuit in Weight Management

The prevalence of obesity in the US has risen in recent estimates to more than 39% [1]. Obesity is associated with an increased risk of diseases with high mortality rates such as coronary heart disease [2]. Modest weight loss can improve the risk of these conditions [3,4]. However, weight loss is challenging, with considerable attrition, perceived barriers, and inconsistencies in outcomes [5,6]. A common approach to facilitate weight loss in behavioral interventions is enhancing goal pursuit, such as helping individuals to form and work towards weight loss goals [7,8,9,10]. Goal pursuit can be understood as “goal setting”, in which users set goals, reflect, and receive feedback on attainment, and “goal striving”, in which individuals make plans to achieve goals and handle barriers [10,11,12,13,14]. Goal setting and striving do not always result in successful goal achievement. Therefore, programs increasingly involve coaches to provide an environment for individuals to explicitly set reasonable goals and cope with emotional challenges in striving to meet them [15,16,17]. So far, despite work suggesting that emotions can have important consequences for weight loss goals, little attention has been paid to individuals’ emotions during goal pursuit. For example, previous work has found that negative emotions predict increased goal-detrimental behavior, such as emotional eating or decreased physical activity [18,19,20]. However, no study has yet explored how individuals’ emotions change during goal setting and striving over the course of weight loss. Further, no study has leveraged computational approaches (e.g., “big data”) [21] to understand this question without relying on self-report.

### 1.2. Computational Approaches to Observe Emotions

Computational approaches through digital technologies such as mobile weight loss programs make possible new insights that are difficult or impossible to collect otherwise [21,22,23]. One particularly relevant large area of research analyzes large amounts of naturally occurring language data in order to understand changes in individuals’ emotions over time [24,25,26,27,28,29]. For example, previous work has gathered user-generated language from social media and has demonstrated that emotional words change over time. Previous studies have found that people reacted with less sadness with increasing time and distance from the Newtown tragedy, or that people “are happier” on weekends than weekdays [27,28], via the emotional words they use on Twitter. Previous work has validated written emotional words as adequate indicators of actual felt emotion [24,30,31]. Though there is some variance in how perfectly the emotional words correlate with felt emotion, emotional words are largely understood to have predictive validity [24,32,33]. This approach is useful because self-report can be subject to recall bias, social desirability bias, and respondent burden [34,35]. In addition, individuals’ self-reported recall of the emotions they felt during a weight loss program can be biased by how much weight they lost [35].

### 1.3. The Current Study

Therefore, this study extends numerous studies measuring emotional language over time to the domain of goal pursuit during weight loss. Specifically, we explore the emotion felt and expressed via emotional words during goal setting and striving conversations with a coach in a mobile weight loss program. Understanding how emotion words change while reflecting on goal progress, particularly when measured in real-time rather than by self-report after finishing the program, can help inform when individuals need extra support to cope with the emotions they are expressing and experiencing. Previous weight loss intervention studies have measured self-reported emotional changes over relatively broad time scales (e.g., 3 or more months [36,37,38,39]), but it is unclear how these patterns will manifest over shorter time spans, such as every month, with unobtrusively measured language. These studies have found that positive emotion increases and negative affect emotion decreases when measured only before and after the intervention. Based on this work, we hypothesize that in goal setting and striving conversations with a coach, positive emotion words will significantly increase over time throughout the program, and negative emotion words will significantly decrease over time throughout the program.

The study’s secondary aim is to explore emotional reactions during goal setting and striving for individuals with varying levels of “goal abstraction”. Goals can be concrete (e.g., “weigh 210 pounds”) or abstract (e.g., “feel good”). Previous work has found that goal abstraction is associated with changes in positive and negative emotion [40,41,42]. According to goal-setting theory, individuals who set specific goals (i.e., “read 20 papers”) experience more positive consequences than abstract goals (i.e., “learn a lot”) [43,44]. This is because individuals are better able to measure progress and break down the steps necessary to attain specific goals [42,43]. Individuals who set abstract goals, however, ruminate about the uncertainty of how to attain the goal, which increases negative emotion [41]. As a result, across many domains, abstract goals are associated with lower positive emotion, higher negative emotion, and decreased goal attainment [11,40,41,43,45]. It is important to note that this discussion of goal abstraction is concerned with overall goals. Individuals can have multiple weight-relevant goals at one time, but some goals are higher-level than others; for instance, one can have a high-level overall goal of weighing 210 pounds and a lower-level, incremental goal of losing 2 pounds per week at the same time [40]. Based on previous studies, we hypothesize that individuals who set an overall concrete goal for their weight loss (concrete goal setters) will show higher positive emotion and lower negative emotion during goal setting than individuals who set an overall abstract goal (abstract goal setters). We also hypothesize that concrete goal setters will have increased goal pursuit and goal attainment than abstract goal setters.

## 2. Materials and Methods

This was a retrospective observational study in which individuals’ language over 4 months in goal setting and striving conversations with a coach were collected after program completion and analyzed from baseline to the end of the program (4 months).

### 2.1. Program

Noom is a mobile behavior change weight loss program that has been found to result in clinically significant weight loss [46,47,48]. Noom is based on cognitive behavioral therapy (CBT), motivational interviewing, and third-wave CBT techniques [49,50,51], each of which aid in weight control. After signing up for the program, individuals are provided with mobile logging features enabling self-monitoring, a curriculum on healthy nutrition, physical activity and behavior change, a virtual group, and a group coach. Each of these components is associated with improved weight management [52,53]. Additionally, as part of the program, individuals have one-on-one text-based conversations with a coach. In these goal setting and striving conversations, coaches help individuals set reasonable incremental goals and consistently ask about individuals’ recent progress, barriers, or difficulties towards the most recent incremental goal. Coaches contact individuals at least once a week. Coaches use goal setting, motivational interviewing, and cognitive behavioral therapy techniques to help individuals to mutually set incremental goals, express their emotions during goal striving, and cope with their emotions and barriers to their goals [17,54]. Goal setting and striving interactions are offered from the beginning to the end of the program. Only user-generated, and not coach-generated, language was used in the study. Transcripts for all participants from the start to end of the program were retrospectively extracted from the program database.

At the start of the program, all individuals are asked to set one overall goal for their weight loss on Noom, with the recognition that they can set smaller incremental goals in their conversations with their coach. Individuals’ overall goals, written at the start of the program, were retrospectively extracted from the Noom database and categorized as “abstract” or “concrete”. Individuals that mentioned a specific, measurable, and/or quantifiable weight loss outcome or process were coded as “concrete” goal setters (e.g., fit into a size 8, lose 15 pounds, be at the weight I was in high school). All other individuals were coded as “abstract” goal setters (e.g., look good, lose belly fat, feel healthy). Krippendorf’s alpha was 0.90, indicating substantial interrater reliability [55].

### 2.2. Participants

**Figure 1:**
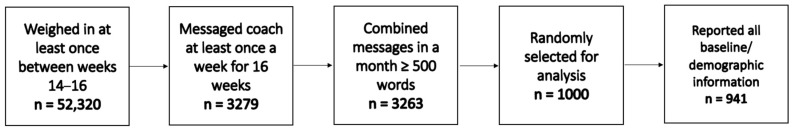
Diagram of inclusion in the study.

Ethical approval for this study was obtained from the Advarra IRB. As part of the approved consent process, all individuals provided informed consent at program sign-up that all their program data could be used for retrospective research. At that time, they were given the option to opt out. To be included in the study, individuals had to be between 18–65 years old, overweight or obese (defined according to federal standards as a body mass index ≥ 25), located in the United States, and signed up during May or June of 2020 [56]. These criteria were chosen to rule out potential confounders (e.g., cultural differences in emotion) and to ensure that all participants had received the full program as intended. In addition, as minimum thresholds for data analysis, participants had to have logged their weight at baseline and at least once any time during weeks 14–16, completed the 16 week program by the time of data collection, messaged the coach at least once a week, and written at least 500 combined words in their messages to the coach each month. Of the individuals who met these criteria, 1000 were randomly selected for the study. Of these 1000, 941 participants provided gender and height at baseline, which were used for BMI calculations, and were included in analyses (see Figure 1). Transcripts of messages to the coach, self-reported weight data, and engagement data for the 941 participants were extracted from Noom’s database (Noom, Inc., New York, NY, USA) and de-identified.

### 2.3. Measures

The most common, well-validated text analysis program, Linguistic Inquiry and Word Count (LIWC) was used for this study [24,57]. LIWC counts the proportion of words in the text that fall in each pre-defined category (or “dictionary”). For example, in a given text, LIWC will count the number of words that are in the “positive emotion” category and provide the proportion of positive emotion words out of the total number of words. LIWC’s dictionary categories are estimated to comprise more than 6000 words [24]. During development, LIWC dictionaries were validated with human coding, and LIWC has since been validated in comparison to self-reported emotion [24,31,32,58]. We used the positive and negative emotion categories from the LIWC dictionary, which are highly correlated with experienced positive and negative emotion [57,59]. All transcripts were combined for each month for each user. In other words, each user had four positive emotion scores: the percentage of positive emotion words used in month 1, month 2, month 3, and month 4. Given that the program focuses on weight loss, goal attainment was measured via changes in weight from self-reported weight measurements on the program. Individuals are encouraged, but not required, to self-report their weight at least once a week. Self-reported weight is a common reliable measure of health behavior goal attainment [60]. We also measured engagement, an indication of how much active effort participants put towards weight loss [61]. Engagement is typically measured via indicators of program activity [62]. Following previous work, engagement was measured by calculating the frequency of the following individual engagement variables: the number of times per week users logged a meal, logged exercise, logged their weight, messaged the coach, read articles, and recorded steps [46].

### 2.4. Statistical Analysis

Linear mixed effects models were used to evaluate changes in language over time and to compare goal abstraction groups. Linear mixed effects models provide more robust parameter estimates than other typical repeated-measures methods [63]. To evaluate changes over time in emotion word categories (e.g., positive emotion words), individual linear effects models predicted each emotion word category using a fixed effect of time (a continuous variable for each month), with a random intercept for each participant and a random slope for time (month). To examine differences in emotion words between concrete and abstract overall goal setters, linear mixed effects models predicted each emotion word category using fixed effects of goal abstraction (abstract vs. concrete goal setter) and time, as well as a random slope for time (month) and a random intercept for each participant. These models included the interaction of time and goal to evaluate differences between groups over time. T-tests were used to evaluate whether there were differences between the goal abstraction groups on engagement and weight. These were not adjusted for baseline weight, BMI, or demographics because there were no significant differences between groups on these variables.

## 3. Results

### 3.1. Baseline Characteristics

Overall, participants had a mean age of 33.44, a baseline weight of 93.39 kg (SD = 20.05 kg), and a baseline BMI of 33.44 (SD = 6.26).

Concrete goal setters (N = 379) and abstract goal setters (N = 562) did not significantly differ on demographic characteristics such as gender, age, height, baseline weight, and baseline BMI (Table 1; all *p*s n.s.). Concrete goal setters were 77.8% female and had a mean age of 47.44, average height of 66.28 inches, mean baseline weight of 94.81 kg (SD = 19.99), and mean baseline BMI of 33.36 (SD = 6.10). Concrete goal setters’ age ranged from 20 to 65 and their baseline BMI ranged from 25.13 to 73.08. Abstract goal setters were 81% female and had a median age of 48.87, an average height of 65.91 inches, a mean baseline weight of 93.9 kg (SD = 20.27), and a mean baseline BMI of 33.45 (SD = 6.47). Abstract goal setters’ age ranged from 20 to 65 and their baseline BMI ranged from 25.03 to 65.26. These demographic characteristics are similar to those found in other weight loss interventions [39].

### 3.2. Descriptive Characteristics of Language

1,587,174 words were analyzed in total. There were 70,175 positive words used and 21,350 negative words used. Positive emotion words were not significantly correlated with weight loss (r(3264) = −0.01, *p* = 0.40), while negative emotion words were marginally significantly correlated with weight loss (r(3264) = −0.04, *p* = 0.10). The more negative emotion words were used, the more individuals lost weight. 

### 3.3. Changes in Emotion Words over Time

We first explored whether positive emotion and negative emotion words significantly increased over time for participants overall (Table 2). The fixed effect of time was not significant in predicting positive emotion words (b = 0.01, *t*(870) = 0.49, *p* = 0.62), which means that positive emotion words did not significantly change over time. However, time significantly predicted negative emotion words (b = 0.05, *t(*876) = 4.71, *p* < 0.001), suggesting that negative emotion words significantly increased over time.

### 3.4. Emotion Words Used by Concrete vs. Abstract Goal Setters

We next explored the emotional words used by concrete goal setters compared to abstract goal setters (Table 3). On average, concrete goal setters used more positive emotion words than abstract goal setters (b = 0.30, *t*(880) = 2.98, *p* = 0.003). The interaction of goal abstraction and time was not significant, suggesting that concrete goal setters and abstract goal setters did not differ in their use of positive emotion words over time (b = −0.07, *t*(802) = −1.7, *p* = 0.09).

In order to rule out the possibility that this finding could be due to concrete goal setters using more emotion words in general, we examined emotional tone. Emotional tone is the ratio of positive emotion words to negative emotion words and is a category in LIWC. If concrete goal setters use more positive emotion words rather than more emotion words in general, they should have higher emotional tone than abstract goal setters. Concrete goal setters indeed had higher positive emotional tone than abstract goal setters (b = 2.60, t(884) = 2.06, *p* = 0.04), which means that they used more positive emotion words relative to the number of negative emotion words they used. The interaction of goal abstraction and time was again insignificant, showing that concrete goal setters did not have a different pattern of emotional tone use over time than abstract goal setters (b = −0.58, *t*(815) = −0.98, *p* = 0.33).

Concrete goal setters did not use fewer negative emotion words on average than abstract goal setters (b = 0.03, t(934) = 0.64, *p* = 0.52). Concrete goal setters did not show different patterns of negative emotion use over time compared to abstract goal setters (b = −0.01, *t*(968) = −0.58, *p* = 0.56).

Concrete goal setters also did not differ from abstract goal setters in program goal attainment, as measured by weight loss (Table 4). Concrete goal setters lost an average of 6.19 kg (SD = 4.64), while abstract goal setters lost 5.99 kg on average (SD = 5.16, *t*(845.6) = 0.63, *p* = 0.53). In addition, concrete goal setters did not significantly differ from abstract goal setters on four of five engagement measures (Table 4). There were no significant differences between the two groups on the number of meals logged (*t*(12,933) = −0.53, *p* = 0.60), exercises logged (*t*(13,309) = 0.68, *p* = 0.49), days with at least one weigh-in (*t*(12,764) = 1.20, *p* = 0.23), messages sent to the coach (*t*(13,392) = −1.50, *p* = 0.13), and steps recorded (*t*(13,267) = −1.60, *p* = 0.11). There were significantly more articles read by abstract goal setters (M = 23.90, SD = 9.49) compared to concrete goal setters (M = 23.24, SD = 10.13; *t*(12,424) = 3.98, *p* < 0.001).

## 4. Discussion

### 4.1. Overall Results

This retrospective observational study was conducted to address several pressing gaps in existing literature. First, there is limited understanding of individuals’ emotions during goal pursuit, though emotional reactions predict behavior that is detrimental for weight loss goals [18,19,20]. Second, ecologically valid measures of real-time emotion during the process of weight loss are rarely used. Previous studies have primarily relied on self-report before and after the intervention, which can be subject to bias both from inaccurate recall and post-intervention outcomes influencing responses. In addition, self-reported measures are limited to specific constructs, which could limit the range of emotions that are captured at one time. On the other hand, computational linguistic analysis is not subject to reporting or recall biases and can reveal intricate relationships that individuals would not be able to self-report, such as the revelation that individuals with depression use more “I” words, representing a higher self-focus [64]. In this study, using a well-validated computerized text analysis program, we retrospectively examined individuals’ emotion language as they reflected on their goals with their coaches in real-time during a weight loss program. This provides a rare glimpse with greater ecological validity into how individuals’ emotional reactions in goal pursuit changed over time during actual use of the program. We found that positive emotion words were stable over time and negative emotion words increased over time. Negative emotion words were marginally significantly associated with weight loss, while positive emotion words were not. We also found that individuals who set an overall concrete goal used more positive emotion words than individuals who set an overall abstract goal. There were no differences between the two groups in negative emotion words, weight loss, or program engagement. Both groups achieved clinically meaningful weight loss (concrete: M = −6.45% body weight; abstract: M = −6.24% body weight) at 16 weeks.

### 4.2. Comparison to Prior Work on Emotion in Weight Loss

We expected that positive emotion words would increase over time and negative emotion words would decrease over time based on past studies that have used pre- and post- measurement of self-reported emotion. These studies found that self-reported positive emotion increased and negative emotion decreased after completing a weight loss intervention [36,37,38,39]. However, via analysis of real-time language, we found that positive emotion words were stable over time and negative emotion words increased over time. It is possible individuals build rapport with their coach and thus felt more comfortable voicing their mounting difficulties over time. This is consistent with the marginally significant trend that more negative emotion words were associated with greater weight loss. The increase in negative emotion is also consistent with prior studies, suggesting that weight management is difficult, especially as time goes on. Individuals face temptations to overeat, social pressures, barriers to their goals, and setbacks such as lapses in eating behavior [6,65,66]. Meanwhile, individuals have to persist in making considerable changes to their diet and behavior, overcoming temptations, facing disappointing results, and mustering the motivation to continue even when they do not see much progress [67,68,69,70]. Additionally, over time, individuals face more conflicts to their weight loss goals and depleted psychological resources, despite early enthusiasm [69,70,71]. These factors can make individuals feel negative emotions such as shame, guilt, frustration, and disappointment [72,73,74]. These qualitative findings suggest that our results could come from the increasing difficulty of sticking to weight loss goals over time. It is also possible individuals built a rapport with their coach and thus felt more comfortable voicing their mounting difficulties over time. Future research should confirm why negative emotion increased over time.

In light of this prior qualitative work, our findings raise new questions for future work. We speculate that our results might show real-time increases in negative emotion over time that were not evident in prior studies using self-reported measures. For instance, perhaps previously demonstrated increases in self-reported positive emotion came from post-hoc realizations that the process was worth it; this speculation aligns with a study showing that people’s recall of their emotions during the program is influenced by how much weight they lost by the end of the program [35]. It is also possible that individuals report more post-study improvement through surveys than they normally might due to social desirability bias. It could also be that language analyses capture broader ranges of negative emotion than common specific indices of depression, anxiety, quality of life, or mood. Future research should compare emotional words during weight loss with self-reported emotions that are measured at regular intervals during the program.

Our results also raise new questions about how emotional words during goal setting relate to behavior. Past studies using electronic momentary assessment (EMA) methods have found that in weight management, negative emotion throughout the course of the day predicts more overeating or less physical activity [20,75]. In addition, while many studies have found consistent relationships between negative emotion and subsequent weight-relevant behavior, fewer studies have found consistent relationships for positive emotion [76]. Our results not only align with this literature but also suggest the possibility that emotional words on this type of program, and in particular negative emotion words, could predict eating lapses or physical activity; this is especially the case since negative emotion words in this study were used in the context of discussing goals. EMA methods require individuals to respond multiple times per time period (e.g., one day) which makes it difficult to use long-term. Therefore, future research should validate and assess the possibility of using emotional words written over the course of the program to predict subsequent behavior. Given that we saw increases in negative emotion over time, future research should investigate whether the use of negative emotions at any given time is predictive or whether changes in negative emotions over time are predictive, or both.

### 4.3. Comparison to Prior Work on Concrete and Abstract Goals

In this study, we also found that concrete goal setters used more language with positive emotion than abstract goal setters. Though individuals can have a number of simultaneous goals, when individuals were asked to choose one overall goal for their weight loss in this program, some wrote an abstract overall goal (abstract goal setters) and some wrote a concrete overall goal (concrete goal setters). There was no difference in negative emotion, goal attainment, and program engagement, except that abstract goal setters read more articles than concrete goal setters. The findings on positive emotion are consistent with literature showing that individuals with concrete goals experience greater positive affect than individuals with abstract goals [42,77]. Our findings on negative emotion diverge from this literature. This could be for several reasons. One possible reason is that abstract goal setters had a clearer sense of how to achieve their overall goals (e.g., “feel healthy”) than one might expect in areas such as general happiness (e.g., “feel happy”), because they could link abstract overall goals (e.g., “feel healthy”) to associated lower-level goals that help with achievement (e.g., “eating more healthy foods”) [41]. It is possible that these links were clearer through the curriculum, which was not solely focused on weight loss but also covered healthy nutrition, lifestyle, and emotional well-being; this could be why abstract goal setters read more articles than concrete goal setters. This is speculative, so future research should elucidate why concrete goal setters did not experience less negative emotion than abstract goal setters.

Our results on similar goal attainment between groups could also be due to a ceiling effect. In order to gather sufficient language data, only participants who wrote at least 500 words each month to their coach were included. It is possible that only highly engaged participants were selected. Engagement is strongly associated with weight loss [78], and both groups lost a clinically significant amount of weight. Therefore, perhaps there was a limit to differences in engagement and weight loss that could emerge between the two groups. Future research should investigate differences in language with a range of engagement and weight loss.

### 4.4. Future Directions and Limitations

Our results raise the question of how and whether language data can be used on a larger scale with big data approaches. While we used a well-validated approach that has been used previously on a larger scale (e.g., millions of words), this is a closed dictionary approach with set language dictionaries [24]. Unsupervised and machine learning approaches make it possible not only to analyze specific frequencies, but also to extract meaningful features of positive and negative emotion, as well as to classify users as who are most and least likely to meet their goals as a result of their language in goal setting and striving conversations [24,79]. Topic modeling approaches could also be used to extract content themes that match changes in emotional words [80]. Future research should explore how to pinpoint individuals in real time who might be in need of additional support through features in language and meta-data.

The study’s limitations include the use of one weight loss program, as well as limited experimental control. Results may not be generalizable to all individuals who seek to lose weight, since results were based on individuals who met inclusion criteria for this study (e.g., messaged their coach each week to enable sufficient data for analysis) and had used this particular program. In addition, in order to unobtrusively analyze data, we did not use self-reported questionnaires of emotions. It is also unknown how the results would apply to individuals who do not write to coaches, due to lack of data.

## 5. Conclusions

**Figure 2:**
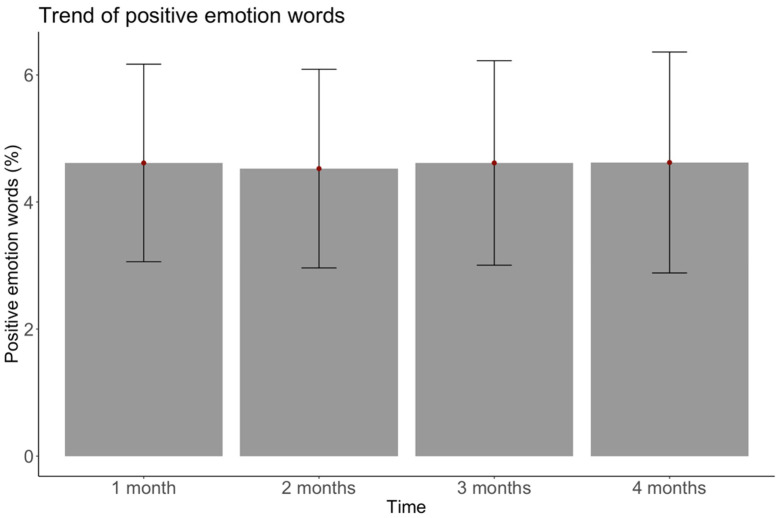
Overall positive emotion words used over time. Note. Bars represent 1 standard deviation above and below the mean.

**Figure 3:**
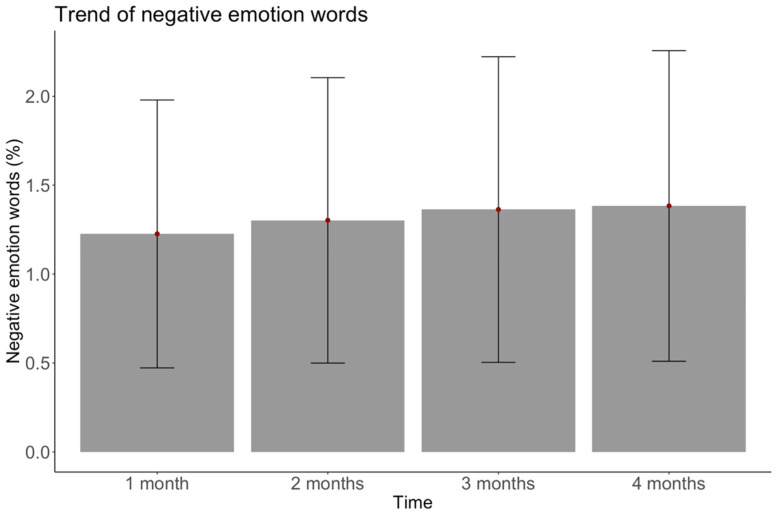
Overall negative emotion words used over time. Note. Bars represent 1 standard deviation above and below the mean.

Researchers increasingly note the promise of computational approaches to “open new doors” for health research [21,81]. Studies using large-scale computational text analysis have previously found that emotional words map onto experienced emotion and reveal new patterns beyond those reported in self-report survey studies, but this approach is rarely used in weight management research [25,26,27,28,29,30,31,82,83]. We extended this work to weight management by collecting over 1,573,000 words used in goal setting and striving conversations on a mobile weight loss program. Using a well-validated computational text analysis program, we found that contrary to self-reports of emotion before and after weight loss programs, real-time negative emotion words during goal setting and striving significantly increased over time, while positive emotion words were stable (see Figure 2 and Figure 3). Positive emotion word usage was high compared to other analyses [84], and remained at a similar level across time. There were fewer negative emotion words used, comparable to other analyses [84,85], and they slightly but significantly increased over time. Additionally, concrete goal setters used more positive emotion words than abstract goal setters, but had similar negative emotion use and goal attainment.

Our findings have implications for creating more effective interventions. Our results suggest that concrete goals could boost positive emotion. Future research should confirm this with experimental designs. Future research should explore whether users were digging deeper into frustrating moments with their coaches over time through increased rapport, or whether they were actually experiencing a greater number of frustrations over time. Regardless, our findings suggest that individuals may need increasing support over time for their increasing negative emotion expression over time. Using more negative emotion words can increase feelings of distress immediately afterwards [86,87,88]. This could be especially problematic for weight management programs since negative emotion predicts emotional eating and reduced physical activity [20,65,76]. As individuals are writing more negative emotional words over time, they may need increasing support immediately after conversations over time. Individuals could also benefit from a greater focus on supportive responses, which help individuals to come to new insights about the negative emotions they shared, decreasing negative emotion and improving well-being [89]. Another implication of our findings is that abstract goal setters could benefit from additional support, such as boosting positive emotion. Positive emotion words are associated with health improvements, such as physical symptoms or physician visits [90], so future research should confirm whether this would improve well-being for abstract goal setters. Overall, the results of this exploratory study suggest many potential implications for more effective support during goal pursuit in weight management, which future research should confirm.

## Figures and Tables

**Table 1:** Baseline Characteristics.

	Goal Abstraction Group		
Variable	Concrete Overall Goal Setters (N = 379)	Abstract Overall Goal Setters (N = 562)	*p*
Age (years), M(SD)	47.44 (11.48)	48.87 (11.28)	0.06
Gender, N(%)			0.25
Male	84 (22.20%)	106 (18.90%)	
Female	295 (77.80%)	456 (81.10%)	
Height (inches), M(SD)	66.28 (3.64)	65.91 (3.46)	0.12
Weight at baseline (kg), M(SD)	94.81 (19.99)	93.90 (20.27)	0.50
Baseline bmi, M(SD)	33.36 (6.10)	33.45 (6.47)	0.83

**Table 2:** Mixed models evaluating changes in emotion words over time.

Predictor	Estimate	95% CI	SE	*t*	*p*
	**Positive Emotion**				
Time	0.01	−0.03, 0.05	0.02	0.49	0.62
	**Negative Emotion**				
Time	0.05	0.03, 0.07	0.01	4.71	<0.001 ***

Note: Linear mixed models investigated whether the fixed effect of time (in months) predicted each emotional language category. Models had a random intercept for each participant and a random slope for time. *** denotes *p* < 0.001.

**Table 3:** Mixed models evaluating differences in emotional word categories between concrete and abstract goal setters over time.

Predictor	Estimate	SE	*t*	*p*
	**Positive Emotion**			
Goal abstraction	0.30	0.10	2.98	0.003 **
Month * Goal abstraction	−0.07	0.04	−1.70	0.09
Month	0.04	0.04	1.43	0.15
	**Negative Emotion**			
Goal abstraction	0.03	0.05	0.64	0.52
Month * Goal abstraction	−0.01	0.02	−0.58	0.56
Month	0.06	0.01	3.81	<0.001 ***

Note. * *p* < 0.05; ** *p* < 0.01; *** *p* < 0.001.

**Table 4:** Goal attainment and program engagement by goal abstraction group.

	Goal Abstraction Group					
Variable	Concrete Overall Goal Setters (N = 379)		Abstract Overall Goal Setters (N = 562)		*t*	*p*
	M	SD	M	SD		
Meals logged per week	27.15	7.92	27.08	7.86	−0.53	0.60
Exercises logged per week	3.22	3.79	3.26	3.92	0.68	0.49
Days with one weigh-in per week	6.06	1.65	6.10	1.61	1.20	0.23
Messages to coach per week	3.64	2.83	3.57	2.96	−1.50	0.13
Steps recorded per week	41,148.37	25,327.68	40,465.28	26,096.11	−1.60	0.11
Articles read per week	23.24	10.13	23.90	9.49	3.98	<0.001 ***
Weight change (kg)	−6.19	4.64	−5.99	5.16	0.63	0.53
Weight change (%)	−6.45%	4.57%	−6.24%	5.45%	0.37	0.53
BMI change	−2.16	1.59	−2.12	1.81	0.40	0.69

Note. *** denotes *p* < 0.001.

## Data Availability

Restrictions apply to the availability of these data. Data was obtained from Noom and are available by request from the corresponding author with the permission of Noom.
